# Supplementary material for: Visiting Urban Green Space and Orientation to Nature Is Associated with Better Wellbeing during COVID-19
Source: Int J Environ Res Public Health. 2023 Feb 17;20(4):3559. doi: 10.3390/ijerph20043559 (PMC9959264; doi:10.3390/ijerph20043559)
Supplement: Supplementary file 1 [file ijerph-20-03559-s001.zip › ijerph-2180904-supplementary.pdf]

Supplementary Table S1. Descriptive statistics of Brisbane and Sydney survey populations.

	**Brisbane**	**Sydney**
PWI (mean, sd)	51.87 (10.70)	50.47 (11.47)
Nature relatedness (mean, sd)	3.54 (0.62)	3.48 (0.60)
Change in wellbeing (count)		
Negative	174	185
Same	661	652
Positive	215	197
Chang in yard visits (count)		
Much less	38	36
Less	99	84
Same	558	474
More	193	221
Much more	64	69
Don’t have yards (na)	98	150
Change in green space visits (count)		
Much less	57	47
Less	110	138
Same	613	585
More	215	228
Much more	55	36
Duration of yard visit last week		
No time	48	73
1–30 min	170	137
31 min to 1 h	131	163
1–3 h	225	229
3–5 h	145	125
5–7 h	83	70
7–9 h	58	36
more than 9 h	92	50
Don’t have a yard or deck	98	150
Frequency of yard visit		
never	24	41
less than once a month	59	60
2–3 times a month	74	81
once a week	144	159
2–3 days a week	205	203
4–5 days a week	190	144
6–7 days a week	256	196
Don’t have a yard or deck	98	150
Frequency of public green space visit		
never	36	43
once a year	36	39
once every three months	97	85
once a month	146	110
2–3 times a month	125	124
once a week	151	164
2–3 days a week	194	187

3–5 days a week	113	138
6–7 days a week	152	144
Duration of public green space visit last week		
Never	277	302
1 h	171	176
2 h	144	112
3 h	87	98
4 h	78	70
5 h	65	48
6 h	29	40
7 h	24	39
8 h	25	18
9 h	16	24
10 or more hours	128	104
NA	6	3
Age group (count)		
18–20 years	35	27
21–25 years	87	83
21–25 years	76	66
31–35 years	90	110
36–40 years	94	97
41–45 years	100	109
46–50 years	84	78
51–55 years	83	89
56–60 years	90	76
61–65 years	90	86
66–70 years	102	92
more than 70 years	119	121
Gender (count)		
Female	505	520
Male	540	511
NA	3	3
Income (count)		
Nil or negative income	10	27
$1–$199 a week	28	21
$200–$299 a week	23	21
$300–$399 a week	40	44
$400–$599 a week	90	83
$600–$799 a week	80	68
$800–$999 a week	80	73
$1000–$1249 a week	104	118
$1250–$1499 a week	91	93
$1500–$1999 a week	130	140
$2000 or more a week	236	200
Prefer not to say	138	146

Supplementary Table S2. The associations between personal wellbeing and nature experiences and nature orientation—sensitivity analysis performed without individuals that indicated they did not have a yard.

	**Estimate**	**SE**	***p***
(Intercept)	34.473	1.952	<0.001
Frequency of yard visit	0.793	0.216	<0.001
Duration of yard visit	−0.223	0.184	0.228
Frequency of public green space visit	0.425	0.139	0.002
Duration of public green space visit	0.053	0.086	0.536
Nature relatedness	1.270	0.480	0.008
Age	0.459	0.081	<0.001
Gender	0.264	0.541	0.626
Income	0.663	0.099	<0.001
City (Sydney)	−0.860	0.525	0.102
